# The Effect of the HLB Value of Sucrose Ester on Physiochemical Properties of Bigel Systems

**DOI:** 10.3390/foods9121857

**Published:** 2020-12-12

**Authors:** Daniel Golodnizky, Maya Davidovich-Pinhas

**Affiliations:** 1 Faculty of Biotechnology and Food Engineering, Technion—Israel Institute of Technology, Haifa 3200003, Israel; dani1994@campus.technion.ac.il; 2 Russell-Berrie Nanotechnology Institute, Technion—Israel Institute of Technology, Haifa 3200003, Israel

**Keywords:** oleogel, hydrogel, bigel, sucrose ester, surfactant

## Abstract

The current research explored the effect of different sucrose esters (SEs), with different hydrophilic–lipophilic balance (HLB) values, on bigel structure and properties. Bigels consisting of a water phase with glycerol and gelatin and an oil phase with glycerol mono-stearate, lecithin, and SEs with different HLB values were prepared. Rheological and thermal analyses revealed similar gelation-melting transitions governed by glycerol-monostearate crystallization (at ≈55 °C) for all bigel samples. The bigel matrix of the H1 and H2 samples (bigels consisting of SEs with HLBs of 1 and 2, respectively) demonstrated physical gel rheological characteristics of higher elastic and solid-like behavior compared with the H6 sample (bigel consisting SE with HLB 6). A similar trend was observed in the mechanical analysis with respect to hardness, firmness, and spreadability values, which were in the order of H1 > H2 > H6. This behavior was attributed to droplet size observed in the microscopy analysis, revealing significantly smaller droplets in the H1 and H2 samples compared with the H6 sample. These differences in droplet size were attributed to the diffusion kinetics of the low-molecular-weight surfactants. More specifically, the ability of mono-esterified SEs to diffuse faster than fully esterified SEs due to lower molar mass leads to a higher SE content at the oil-in-water (O/W) interface as opposed to the bulk oil phase. The results demonstrate the importance of the interface content in O/W bigel systems, providing an effective way to alter and control the bigel bulk properties.

## 1. Introduction

The food industry, as well as the cosmetics, pharmaceutical, and agricultural industries, produce large amounts of emulsion-based products, such as vinaigrette, mayonnaise, milk products, and various spreads [1,2]. Emulsions, or biphasic systems, exhibit a wide range of physiochemical and sensory characteristics depending on their composition and processing conditions used in their production [2]. The texture of a biphasic system may be altered by structuring the oil or water phases, forming either a gel-filled emulsion or a bulk gel emulsion in which either the dispersed or the continuous phase is gelled, respectively [3]. Recent studies explored the ability to structure both phases, leading to the formation of a hydrogel, gel based on water as a continuous phase, which is usually stabilized by a polymer cross-linked network, and an oleogel, gel based on oil as a continuous phase, which is stabilized using oil structuring agents such as mono acyl-glycerols, which then are combined to form a final gel product termed a bigel. Such a combined system may result in hydrogel drops dispersed in oleogel continuous phase, oleogel drops dispersed in hydrogel continuous phase, or bi-continuous organization. Bigels showed high potential as drug delivery systems due to their ability to possess the characteristics of both hydrogels and oleogel [4,5]. Two different preparation procedures were suggested that differ in the state of the phases during mechanical stirring. The first method involves direct mixing of the two gels at room temperature [4,5,6,7], while according to the other approach, the mixing process is performed with both phases in liquid state [8,9]. The first approach does not require the addition of an emulsifier during preparation due to the solid state of the phases. Such an approach potentially limits the system stability over time due to the absence of surface-active molecules responsible for the interface stability. The second approach relies on the ability of a surface-active agent to stabilize the water/oil interface of two immiscible liquids. This approach provides a way to control the bigel’s final properties by controlling emulsion characteristics such as droplet size and distribution, which are directly related to the surface-active agent type and concentration.

Emulsion stabilization is strongly dependent on the presence of surface-active agents, which are also known as emulsifiers. Emulsifiers adsorb to the surface of the droplets, forming a protective layer that prevents droplet coalescence and aggregation, thus stabilizing the system [2,10]. Emulsifiers are classified primarily based on their molecule size (i.e., small and large), while an additional sub-classification is based on surfactant charge [11]. The droplet size and distribution are determined by the ratio between the droplet collision rate and the rate of emulsifier adsorption to the interface [12]. The emulsifier adsorption rate is strongly related to its molecular size whereby small molecules, such as lecithin, adsorb to the water/oil interface faster than large molecules, such as proteins and polysaccharides [2,10]. Combining emulsifiers can change the stability and functional properties of emulsions. For example, combinations of small surfactants, which adsorb rapidly to the interface but do not provide the coalescence with good long-term stability, and large molecular architectures, such as proteins, which adsorb slowly but provide good long-term stability [13] [12]. Another important aspect of emulsifier functionality is the hydrophilic–lipophilic balance (HLB), which is proportional to the ratio of polar to nonpolar sites of the molecule, while molecules with high hydrophobic content will have lower HLB values. An HLB value strongly affects the emulsion type, i.e., water-in-oil or oil-in-water, and its stability [14]. A higher proportion of nonpolar sites, resulting in a lower HLB value, will usually result in water-in-oil emulsion, while a lower proportion of polar sites, resulting in higher HLB values, will usually result in oil-in-water emulsion [13,14]. The ability of surfactants with different HLB values to absorb to the interface in a system that combines more than one emulsifier will be affected by the emulsifier HLB value and concentration. For example, Moran-Valero et al. [11] showed that while combining lecithin and glycerol mono-stearate (GMS) to stabilize O/W emulsion, the GMS, which exhibits a lower HLB value compared with lecithin, was found inside the oil droplets, while lecithin was found on the interface. Such a phenomenon was attributed to the preference of a high HLB value emulsifier to stabilize the oil-in-water emulsion interface. Other effects are related to the water/oil ratio used and the emulsifier concentration, which both affect the emulsifier water/oil interface coverage. For example, Ojeda-Serna et al. [15] examined the effect of different water/oil ratios on particle size and rheological characteristics of water-in-oil (W/O) oleogel-based emulsions. Pichot et al. [16] examined the effect of the emulsifiers’ molecular weight and concentration on characteristics of O/W emulsions using Tween 60, sodium caseinate, lecithin, and mixtures thereof. In their research, they showed that increasing the concentration using a single-type emulsifier led to a decrease in droplet size up to a certain concentration after which no further decrease was observed. Moreover, combining low and high molecular weight emulsifiers provided both short- and long-term stability by avoiding both coalescence and an increase in droplet size.

A study recently conducted in our lab explored the formation of bigel systems based on gelatin hydrogel, glycerol mono-stearate (GMS) oleogel, and lecithin using a hot emulsification procedure with various compositions and preparation conditions [17]. The current research further explores this system by manipulating the surfactant type and characteristics, with the objective of controlling emulsion properties that affect the final bigel properties. To this end, bigels were formulated using gelatin and GMS as structuring agents of the water and oil phases, respectively. The water/oil ratio was 1:1, and sucrose ester (SE) was added as a surfactant with different HLB values as well as a minimum amount of lecithin. 

Gelatin hydrogels are formed in a temperature-induced crosslinking process driven by the formation of gelatin helices followed by their self-assembly into a three-dimensional branched network [18,19]. The process of structuring oil using GMS is controlled by the molecular self-assembly of GMS into lamellar structures, which further aggregate into a microscale crystal network [20,21]. Soy lecithin contains a variety of phospholipids that are extracted from oil-bearing seeds of soybean. They are well-known surface-active agents with an HLB value of approximately ≈7 and are used widely as emulsifiers in a vast range of foods, as well as pharmaceutical and cosmetic applications [22,23]. SEs are a family of surfactants derived from the esterification of fatty acids to sucrose molecules. As sucrose contains eight hydroxy groups, SEs with mono- to octa-fatty acid esters can be found. SEs are non-ionic lipophilic surfactants in which the sucrose segment acts as a hydrophilic group and the fatty acids act as lipophilic groups. By changing the number of fatty acids esterified to the glucose ring, surfactants with a wide range of HLB values may be obtained [24]. In the current research, we investigated the effect of SEs with different HLB values on bigel characteristics. Determining the relationship between the surfactant’s characteristics, its emulsifying ability as expressed by its emulsifying capacity and stability, and the final emulsion gel functionality is a very interesting challenge that can be used for future development of these types of bigels in order to manipulate food texture in various applications.

## 2. Materials and Methods

### 2.1. Materials

Soy lecithin was purchased from Texturot (Israel). SEs: SP01 (HLB = 1), SP10 (HLB = 2), and SP30 (HLB = 6, also referred as food additive E473) were kindly donated by Sisterna (Netherlands, via local distributor Nagum, Israel). Glycerol monostearate (GMS), which is sold under the commercial number 0091, was kindly donated by Palsgaard (Denmark). Glycerol anhydrous was purchased from Bio-Lab Ltd. (Israel). Silicone oil was purchased from Sigma-Aldrich (Israel) and gelatin was purchased from BD Bioscience (Israel). Canola oil was purchased from the local supermarket (private label Shufersal).

### 2.2. Emulsion Preparation

Bigels were prepared in 60-g batches with a 1:1 water/oil ratio, where the water phase consisted of 28.2 g water, 1.5 g glycerol, and 0.3 g gelatin, and the oil phase consisted of 21.3 g canola oil, 7.5 g GMS, 0.6 g SE (SP01, SP10, or SP30), 0.48 g lecithin, and 10 ppm silicon oil. The water phase and oil phase were stirred separately and heated, respectively, to 70 and 90 °C on a hot magnetic plate stirrer (IKA- Werke GmbH & Co. KG, Staufen, Germany) until full dissolution was reached before being combined. The still-hot oil phase was added manually, dropwise, to the still-hot water phase, followed by homogenization at 16,000 rpm for 1 min using an Ultra-Turrax handheld homogenizer (Miccra D-9, MICCRA GmbH, Heitersheim, Germany). After homogenization, samples were stored at 4 °C overnight before further examination. 

### 2.3. Rheological Measurements

The bigels’ rheological properties were analyzed using an MCR 302 rheometer (Anton Paar, Prime Lab Scientific, Kfar-Saba, Israel) with parallel plates (40-mm diameter) and a 1-mm gap. The input amplitude strain used for the dynamic analysis was 0.1%, which is a value that was found to be within the linear viscoelastic region determined in preliminary experiments. A temperature sweep test was performed to monitor the sol–gel transition process that occurs during cooling. A temperature ramp from 80 to 10 °C using a 5 °C min^−1^ cooling rate at ω = 10 s^−1^ and a 0.1% shear strain was used. A frequency sweep experiment was conducted to analyze the gel viscoelastic properties aiming to reveal the gelation mechanism and gel properties. A frequency sweep test between 0.01 rad·s^−1^ and 100 rad s^−1^ at a fixed strain of 0.1% was used. The flow behavior of the samples was analyzed by measuring the viscosity with respect to shear rate from 0.01 to 100 s^−1^ using a logarithmic ramp, which provides additional information regarding the bigel flowability. All rheological measurements were performed in triplicate.

### 2.4. Thermal Analysis

Thermal analysis was performed using differential scanning calorimetry (DSC) and thermogravimetric analysis (TGA) in order to analyze the thermal transition occur during heating and the involvement of bounded and unbounded water in this process. DSC analysis was performed using a DSC 250 apparatus (TA Instruments, New Castle, DE, USA). The samples were weighed in aluminum pans and heated from 40 to 200 °C at a rate of 3 °C min^−1^ under nitrogen at a flow rate of 50 mL min^−1^. TGA analysis was performed using a TGA 5500 apparatus (TA Instruments, New Castle, DE, USA) under nitrogen. The samples were weighed in platinum pans and heated from 40 to 700 °C at a rate of 10 °C min^−1^. Thermal analysis was performed in triplicate.

### 2.5. Confocal Microscopy

The bigel microstructure was analyzed using inverted confocal laser scanning microscopy (CLSM 710, Zeiss, Oberkochen, Germany). This technique was used to reveal the emulsion type obtained and the size and distribution of the emulsion droplets. The images were taken after staining the oil phase with Nile red (excitation at 488 nm) and the gelatin in the water phase with Nile blue A (excitation at 633 nm) using a final concentration of 0.05 µL mL^−1^ stain (stain to phase ratio) [25]. Microstructural analysis was done with a 20× objective and a 2 µm pinhole diameter. The images were taken and analyzed for particle size and distribution using ImageJ program, and *d*_43_, *d*_32_, and *c*_2_ were calculated using the following equations:

(1) $$d_{43}\;=\;\frac{\sum n_{i}\times d_{i}{}^{4}}{\sum n_{i}\times d_{i}{}^{3}}$$

(2) $$d_{32}\;=\;\frac{\sum n_{i}\times d_{i}{}^{3}}{\sum n_{i}\times d_{i}{}^{2}}$$

(3) $$c_{2}\;=\;{\left(\frac{\sum (n_{i}\times d_{i}{}^{2})\times \sum (n_{i}\times d_{i}{}^{4})}{{\left(\sum n_{i}\times d_{i}{}^{3}\right)}^{2}}\;-1\right)}^{1/2}$$

where *d*_43_ is the volume–length diameter, *d*_32_ is the area–volume diameter, *c*_2_ is the relative standard deviation weighted with the 2nd power, *d_i_* is the diameter of the particle, and *n_i_* is the number of particles in each size class [2].

### 2.6. Texture Profile Analysis

Textural properties, namely hardness and cohesiveness, were evaluated after samples were stored at 4 °C using the texture profile analysis (TPA) procedure and a TA1 texture analyzer (Lloyd Instruments Ltd., West Sussex, UK). TPA analysis was used to evaluate the effect of different SEs on the bigel’s textural attributes, which can lead to different emulsion applications and consumer experience. The sample was compressed twice between two cylindrical plates (11.5 cm diameter) to 75% of its original height using an overhead speed of 90 mm min^−1^ and a 50 N load cell. Samples were prepared as described above, poured into a 60 mm aluminum weighing dish, and allowed to rest at 4 °C for an hour to crystallize and stabilize. After crystallization and stabilization, 19 mm pucks were extracted from the center of the dish using a cylindrical cookie cutter and stored overnight at 4 °C before further analysis. Force–extension curves were recorded, and the maximum force obtained during the first compression cycle (or bite), which expresses the hardness, was calculated. We also calculated the ratio of work done during the first bite divided by the work done during the second bite, which expresses the cohesiveness. The textural properties of oleogels containing the oil phase components, i.e., GMS, lecithin, SE, and oil, were examined using the same procedures described above. All values were calculated based on at least six replicates.

### 2.7. Spreadability

The effect of bigel composition on the spreadability of the gel was analyzed using a spreadability jig (90° cone probe and sample pots) mounted on a TA1 texture analyzer (Lloyd Instruments Ltd., West Sussex, UK). Bigel samples were prepared using the method described above, poured directly into the spreadability pot, and allowed to set overnight at 4 °C before testing. The probe penetrated 15 mm into the sample at an overhead speed of 50 mm min^−1^, while the normal force was measured. Firmness and spreadability were calculated as the maximum force during penetration and the work done during the test, respectively. Values were calculated based on at least six replicates.

### 2.8. Statistical Analysis

Statistical analysis was performed using GraphPad Prism 7.0 (GraphPad software, San Diego, CA, USA). Tukey’s multiple comparison test was conducted. Each sample was prepared and measured at least three times.

## 3. Results and Discussion

### 3.1. Emulsion Preparation

Bigels were prepared using the formulation previously described with some modifications [17]. Table 1 presents the compositions of the different samples. The total concentration of SE used, i.e., 1 wt %, is the maximum concentration permitted by the Israeli regulation on food additives issued by the Israeli Ministry of Health. This concentration of SE was not enough to stabilize the bigel emulsion by itself, and so lecithin was also added at the minimum concentration needed, i.e., 0.8 wt % (determined separately). Glycerol was added to the water phase as a co-surfactant [26] and, finally, 10 ppm silicon oil was added to minimize foaming during homogenization [27]. During this work, it was assumed that this low silicon oil concentration did not affect interactions between the surfactants at the water/oil interface.

### 3.2. Analysis of the Gelation Process Using Rheology

**Figure 1:**
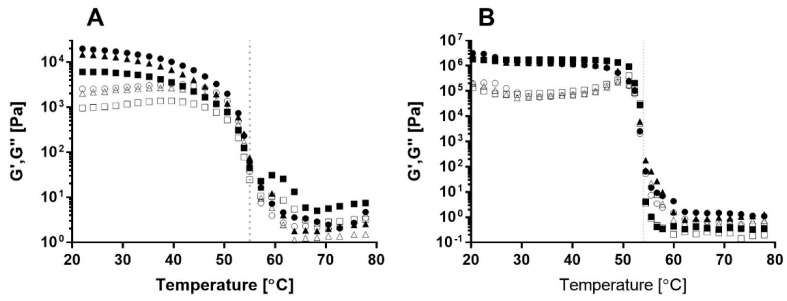
Typical temperature sweep moduli curves obtained for (**A**) bigel and (**B**) oleogel produced with SE HLB1 (●,о), HLB2 (▲,∆), and HLB6 (■,□) at 5 °C min^−1^ cooling rate. Storage modulus (G’, closed symbols) and loss modulus (G’’, open symbols). Oleogel samples consist of 25 wt % GMS, 1.6 wt % lecithin, and 2 wt % SE.

The gelation process of the different bigels with different SEs was monitored by temperature-dependent dynamic oscillatory rheological measurements (80 °C–20 °C, see Figure 1A). In this analysis, the viscoelastic properties of the sample were expressed by (a) the storage modulus, G’, which is defined as the elastic characteristic of the sample and represents the solid-like behavior of the material, and (b) the loss modulus, G’’, which is defined as the viscous characteristic of the material and represents the flow and mobility of the sample [28]. A clear transition from viscous liquid-like behavior, characterized by G’’~G’, to solid-like behavior, characterized by G’>G’’, is evident during the cooling process of the samples [29]. The sol–gel transition temperature or characteristic gelation temperature was recorded as the temperature at which G’ rapidly increased during the cooling process and a significant difference between the G’’ and G’ was detected, with G’>>G’’ [30].

A gelation temperature of approximately 55 °C was recorded for all bigel formulations and was reflected by a sharp increase in both moduli during cooling (Figure 1A). GMS crystallization in oil occurred around 40–60 °C, depending on oil type, GMS concentration, and gelation conditions [20,21,31], and gelatin gelation in water occurred around 18–35 °C, depending on gelatin concentration and setting temperature [32]. Moreover, a previous study reported lower gelation temperatures in the range of 25–55 °C for GMS oleogels prepared using various concentrations in the range between 0.75 and 8 wt % [33]. These results suggest that the sol–gel transition detected in the current analysis is governed by GMS crystallization rather than by gelatin gelation.

Similar gelation temperatures were observed for the different samples with various SEs, suggesting that the emulsifier’s HLB value did not affect the gelation process, as expressed by the gelation temperature. Bin Sintang et al. [34] studied the gelation of oil using combinations of SEs and lecithin and found that a decrease in the SE:lecithin ratio shifted the gelation transition of the oleogel to a lower temperature. This behavior was attributed to interactions between SE and lecithin and their organization in the oil phase. To evaluate the involvement of SE in oleogel stabilization and its effect on bigel gelation, the oil phase content, i.e., the oleogel, was examined using the same procedure (Figure 1B). As can be seen, the sol–gel transition of the oleogels with different SEs occurred at the same temperature, suggesting that the HLB value does not affect the gelation temperature of the oil phase. Moreover, the gelation temperatures of the oleogel and bigel are similar, supporting our assumption that bigel gelation is governed by GMS crystallization, leading to oil phase stabilization. It is interesting to note that the gelation transition of the oil phase (Figure 1B) appears as a sharp, distinct transition, while the gelation transition of the bigel (Figure 1A) is more moderate. This can be attributed to the presence of a water phase in the bigel that might interfere with the gelation process.

Thus, it can be concluded that the bigel gelation detected in the temperature sweep analysis is governed by the gelation of the oil phase, i.e., GMS, and that it is unaffected by the HLB value of the SE. 

### 3.3. Thermal Analysis

The gelation thermal transition was also monitored using DSC in order to verify the involvement of the GMS crystallization in the bigel gelation as suggested in the temperature-dependent rheology analysis. More specifically, the melting temperaure of the oil phase and the evaporation temperature of the water phase were determined using DSC. 

**Figure 2:**
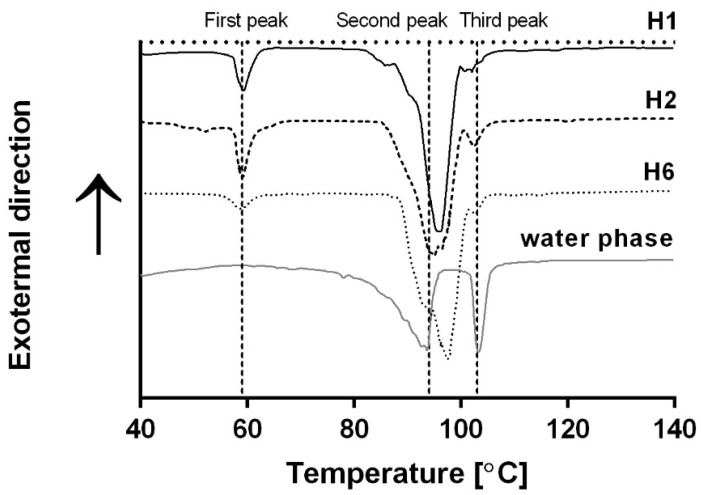
Differential scanning calorimetry (DSC) thermogram (3 °C min^−1^) of H1 (black curve), H2 (dashed black curve), H6 (dotted gray curve), and the water phase (gray curve).

The heat flow required to heat the samples using a 3 °C min^−1^ heating rate demonstrated three main endothermal peaks (Figure 2). The first peak, at ≈59 °C, can be related to the melting temperature of GMS in oil [35]. This peak was observed only in the bigel thermograms and was absent from the water phase thermogram, supporting our assumption. No difference was found in the location of the first peak in the thermograms of the different bigels, suggesting that the esterification degree of the SE had no effect on the melting temperature of GMS crystals in the oil phase. This observation is in line with the results of the temperature sweep analysis, suggesting that the SE type does not affect the oil phase sol–gel transition.

Two additional endothermic peaks were identified at ≈94 and ≈103 °C, respectively. The melting temperature of gelatin hydrogels ranges between 10 and 40 °C [18], while the evaporation temperature of glycerol is about 290 °C [36]. Therefore, these peaks were attributed to the evaporation of water from the bigel system. The water dehydration process can be divided into two steps: dehydration of free water and dehydration of bound water. The differences between these two processes relate to the temperatures at which the water molecules dehydrate, whereby free water dehydrates at a lower temperature compared with bound water molecules [37]. Therefore, the second and third peaks were associated with the free and bound water within the water phase, respectively. Bellich et al. [38] characterized the evaporation behavior of aqueous alginate solutions to which different molecules were added. In the study of Bellich et al., a single endothermic peak was detected for the water evaporation process, which was characterized by an exponential increase in heat flux as a function of temperature, ending in a maximum followed by a sharp decrease. The effect of different solutes on the peak maximum temperature and the sharp decrease observed were related to the ability of each solute to bind water. A similar water hydration process, characterized by one endothermic peak with peak broadening due to solute content, was observed in water/poly(vinyl alcohol)/glycerol systems [39]. In the current research, two separate distinct endothermic peaks were detected for the free and bound water. It seems that the heating rate used in the current research, i.e., 3 °C min^−1^, as opposed to the heating rates used in the above studies, i.e., 5 and 10 °C min^−1^, yielded better separation between the peaks of the bound and unbound water.

The presence of two distinct peaks reflecting water evaporation was seen in all samples, i.e., water phase containing gelatin, glycerol, and water, and different bigels. However, the bound water peak, i.e., the third, high temperature peak, was broader and smaller in the bigels compared with the water phase samples. This phenomenon can be attributed to gelatin diffusion to the water/oil interface during emulsification, which acts as a long-term stabilizer of the emulsion [13]. The water phase analysis (without the presence of the oil phase) examines hydrated gelatin molecules in the water phase, and so maximum water molecules are bound to the polymer backbone. The results in Figure 2 suggest that combining oil and water phases, such as in the case of the examined bigels, leads to a decrease in the amount of bound water, which can be the result of a decrease in the amount of fully hydrated gelatin molecules. Such behavior can be related to the diffusion of gelatin molecules to the water/oil interface, decreasing the amount of hydrated gelatin molecules in the water. Thus, less water molecules were bound to the gelatin and the magnitude of the third peak decreased.

**Figure 3:**
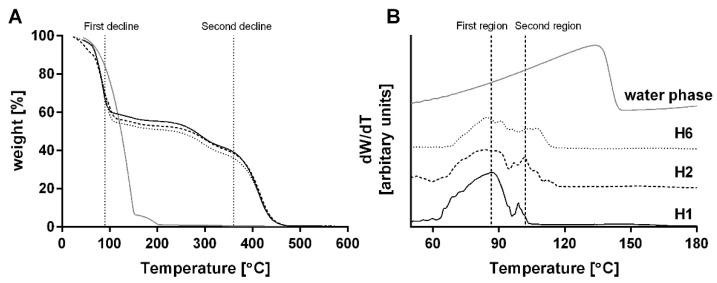
Thermogravimetric analysis (TGA) of H1 (black curve), H2 (dashed black curve), H6 (dotted gray curve), and water phase (gray curve) using a 10 °C min^−1^ heating rate. (**A**) Percentage of weight as a function of temperature and (**B**) derivative of weight percentage by temperature as a function of temperature.

To further examine the hypothesis that the decrease in the third peak seen in the DSC thermograms (Figure 2) resulted from a decrease in the amount of bound water in the bigel compared with the water phase, TGA was performed. The bigels and water phase, consisting of glycerol and gelatin, were heated while being weighed to estimate the amount of bound and free water in each sample. Typical weight loss and derivative of weight loss thermograms are presented in Figure 3A,B, respectively. Figure 3A presents two main weight loss regions, the first around 90 °C and the second around 360 °C, as well as a smaller weight loss region around 300 °C. The typical boiling temperature of vegetable oils is around 390 °C [40], and so the higher temperature weight loss region (≈360 °C) was attributed to the evaporation and decomposition of the oil phase. The smaller weight loss region around 300 °C was attributed to the decomposition of gelatin and glycerol, as determined by TGA measurements done on the pure components. The decomposition of proteins [41] in general, and specifically of gelatin and glycerol [42], were previously detected at this temperature range. Finally, the first weight loss region, around 90 °C, can be related to water evaporation [38]. This water dehydration step was found to be ≈40 wt % out of the bigel samples (Figure 3A), which is close to the percent of water used to prepare the bigels, i.e., 47% (Table 1). Moreover, the main weight loss of the water phase consisting of gelatin, glycerol, and water (gray line) occurred in this temperature range, supporting our assumption that this region in the bigel thermograms is related to water dehydration. Thus, to examine the behavior of the bound and free water in the bigel samples, the analysis concentrated on this region. Figure 3B presents the derivative of the percentage of weight loss (derivative thermogravimetry, DTG) with temperature, as a function of temperature, between 50 and 180 °C, for the different samples. The curve obtained from the water phase (gray line) has a single peak with a maximum at ≈135 °C. The onset temperature of this peak is around 30 °C, and the evaporation curve increases gradually from this temperature until it reaches a maximum followed by a sharp drop. This behavior is characteristic of the water evaporation process seen in TGA [38,39]. The location of this peak maximum at such a high temperature suggests that the water molecules in the water phase are bound, as suggested also by the DSC analysis. The DTG curves for the bigels exhibited two main temperature regions: the first at a lower temperature, ≈86 °C, which can be associated with free water, and the second at a higher temperature, ≈102 °C, which can be associated with bound water. These results are in line with the DSC analysis presented above. It is interesting to note that the magnitude and area of the second peak (≈102 °C) are significantly lower than those of the first peak (≈86 °C), suggesting that the mass fraction of the bound water molecules in the bigel formulation is lower than that of the unbound molecules. Moreover, the presence of two regions in the bigel curves, as opposed to a single region in the water phase curve, suggests that the polymer in the water phase is fully hydrated, while in the bigel, there is a distinct difference between the protein in the bulk water and the protein at the interface. Thus, it seems that part of the water that was bound to the protein in the water phase became unbound after mixing and homogenization with the oil phase. This result supports our assumption that gelatin diffuses to the water–oil interface in bigel systems and thus can potentially affect the emulsification and stability of the bigel system, which will be further discussed below.

### 3.4. Viscoelastic Properties

**Figure 4:**
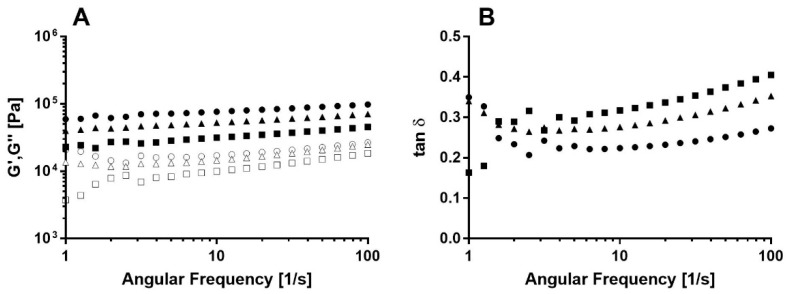
Typical (**A**) frequency sweep curves representing the storage modulus (G’, closed symbols) and loss modulus (G’’, open symbols) of various bigel samples for H1 (●,о), H2 (▲,∆) and H6 (squares) bigel samples, HLB2, and HLB6 (■,□). (**B**) the corresponded calculated tan δ for H1 (●), H2 (▲) and H6 (squares) bigel samples, HLB2, and HLB6 (■).

The gel’s viscoelastic properties were determined to examine the gelation mechanism and gel type. Frequency sweep tests were applied to various formulations, and the results are presented in Figure 4. All samples exhibit significantly higher G′ values compared with G′′ over the entire frequency range, suggesting solid-like gel behavior [29].

Gels can be classified into two main categories: weak physical gels, in which G′ and G′′ depend on the frequency, and strong chemical cross-linked gels, in which G′ and G′′ are relatively frequency-independent [43]. The results show a slight frequency dependency, as evidenced by the weak positive slope of G′ and G′′ vs. frequency curves. This dependency can be expressed using a power-law model [44].

(4) $$G^{\prime }={\;G}_{0}^{\prime }\cdot \omega^{\;n\prime }$$

(5) $$G^{\prime \prime }={\;G}_{0}^{\prime \prime }\cdot \omega^{\;n\prime \prime }$$

where $G_{0}^{\prime }$ and $G_{0}^{\prime \prime }$ are, respectively, the storage and loss moduli at 1 rad·s^−1^, n′ and n′′ are, respectively, the exponent expressing the frequency dependence of G′ and G′′, and ω is the angular frequency in s^−1^.

Table 2 summarizes the fitted parameters from Equations (4) and (5). In general, all bigel samples exhibited positive frequency-dependent moduli values expressed by an exponent value greater than zero. Such a tendency implies the physical character of the gel, which dictates a change in moduli with frequency [45]. In general, lower G′ exponent values, n′, were detected compared with the G′′ exponent value, n′′, which is also typical for physical gels [46]. Exponent values in the order of H1 < H2 < H6 were obtained for both moduli. Higher exponent values imply frequency-dependent behavior, suggesting that the gel network is more sensitive to applied shear. The loss and storage moduli at 1 rad·s^−1^, i.e., $G^{\prime }$ and $G_{0}^{\prime \prime }$, demonstrated opposite trends, with values in the order of H1 > H2 > H6. It is well established that G′ expresses the solid-like behavior of viscoelastic materials, and so a higher G′ value implies a harder gel. Therefore, it can be concluded that H1 and H2 samples exhibited harder mechanical characteristics compared with H6 and that H1 was the hardest bigel while H6 was the softest. A similar trend was seen in the loss and storage modulus at 1 rad·s^−1^ of Surimi gels when the polymer concentration was increased, leading to harder gels [47].

Analysis of the loss and storage moduli ratio, termed the loss factor, i.e., tan δ = G′′/G′, provides an additional point of view on the viscoelastic behavior of the gels. In the frequency range tested, all samples exhibited solid-like characteristics, with tan δ < 1 [2]. However, a positive frequency-dependent slope was observed, suggesting a moderate softening transition with frequency to a more liquid-like behavior. This trend was more significant for the H6 sample compared with the H2 and H1 samples, as can be seen by the change in the curve slopes (Figure 4B). The relationship between gel strength and loss factor value was also observed previously by Ojeda-Serna et al. [15], while analyzing the effect of water content on the viscoelastic properties of W/O myverol oleogel-based emulsion with coconut and canola oils. In this study, higher loss tangent values were obtained when using a lower concentration of water, leading to more solid-like characteristics at lower water concentrations.

In conclusion, bigels produced with SEs with low HLB values exhibit higher elastic properties with more solid-like behavior compared with bigels produced with higher HLB SEs. To further understand the source of this trend, particle size analysis using microscopy was performed.

### 3.5. Microstructural Analysis

**Figure 5:**
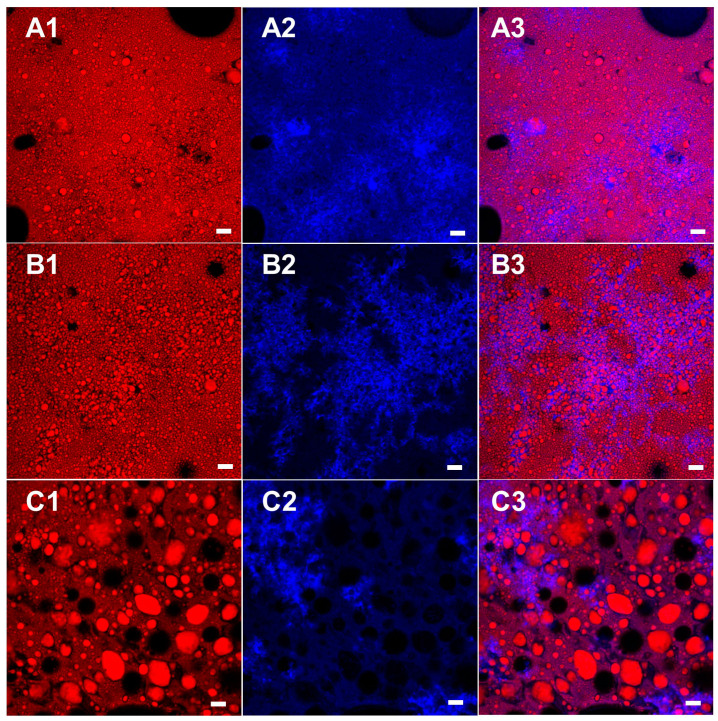
Confocal micrograph obtained for bigel samples using 20× objective (A) H1; (B) H2; and (C) H3 stained with Nile red (oil phase) and Nile blue A (gelatin in the water phase). (1) Oil phase, (2) water phase, and (3) combined image of both phases. Bar = 20 µm.

The microstructure of the different bigel formulations obtained using three different types of SEs was studied using confocal microscopy (Figure 5). This technique was used to determine the location of each phase, i.e., water and oil, and to analyze the droplet size and distribution. In all three formulations, oil droplets (red) were observed within the continuous water phase (blue), verifying the formation of O/W emulsions. Emulsions can be classified according to the relative spatial distribution of the oil and water phases into W/O or O/W emulsion types [2]. The HLB value of surfactants can provide a useful indication as to which emulsion it will form; low HLB surfactants (3–6) favor the formation of W/O emulsions, while high HLB surfactants (10–18) favor the formation of O/W emulsions. Surfactants with intermediate HLB values (7–9) have no particular preference [2,48]. In the current research, we used lecithin, which has an HLB of ≈7, GMS with an HLB around ≈3.8, and three different SEs with HLB values of ≈1, 2, and 6. Based on these HLB numbers, one would expect to obtain W/O emulsions, but it seems that the combination of the different surfactants led to the formation of an O/W type emulsion. Lecithin has a higher HLB value than all of the SEs, and so it was able to stabilize smaller droplets in O/W emulsions [11,13]. Moreover, it was shown that a combination of two or more surfactants with different HLB values can be used to create a system with a specific HLB value, thus controlling the formation of a specific emulsion type [49].

GMS was used in the current system as an oil-structuring agent, although its high concentration and emulsification properties can potentially direct some of the GMS molecules to the water–oil interface. A previous study focused on a combination of lecithin and GMS as emulsifiers in water–oil systems and showed the formation of an O/W type emulsion [11]. The bigel system also contains gelatin molecules aimed at gelling and stabilizing the water phase, which can also participate in the stabilization of the water–oil interface as discussed in the thermal analysis section (Section 3.2). The ability of protein to stabilize the water–oil interface is driven by the presence of both hydrophobic and hydrophilic amino acids. This tendency is even stronger when the protein solution temperature is increased, exposing the hydrophobic areas of the protein, which can potentially lead to absorption at the oil droplet interface. The bigel preparation procedure discussed in the current research involved hot emulsification at 70 °C, which can potentially promote protein unfolding and absorption at the water–oil interface due to the high temperature [50] and the homogenization process [51]. The ability of gelatin to stabilize O/W emulsions was examined previously; however, relatively low surface activity was suggested for fish gelatin compared with globular proteins such as β-lactoglobulin [52]. Therefore, it can be concluded that despite the use of surfactants with low or intermediate HLB values, which usually favor the formation of W/O emulsions, the presence of gelatin and GMS led to the formation of O/W emulsions.

The micrographs demonstrated the effect of the different SEs on particle size and distribution, where H1 and H2 formulations demonstrated similar particle size that was relatively smaller compared with the H6 sample. Droplet size and distribution, based on the d_32_, d_43_, and C_2_ values, were determined by analyzing the image of each formulation using ImageJ software (Table 3). The droplet sizes d_32_ and d_43_ represent the surface-weighted mean diameter and volume-weighted mean diameter, respectively, while C_2_ represents the relative standard deviation. In general, among the common ways to express the mean particle diameter of polydisperse emulsions, it can be said that the higher the order of the mean (a + b sum in d_ab_), the higher the numerical value and the more significant the weight of every larger droplet present, increasing the mean [2]. The results confirm similar d_43_ and d_32_ values for H1 and H2 samples, which are significantly smaller than the values obtained for the H6 sample. The large differences in mean particle size values may be indicative of large polydispersity, which is demonstrated by the C_2_ value. C_2_ values range from 0.1 for a very narrow droplet distribution to 1.3 for a very wide distribution [2]. A narrower particle size distribution will result in comparable d_43_ and d_32_ values, as can be seen for H1 and H2, while for wide distributions, such as in the case of H6, d_43_ values are higher compared with d_32_, as are C_2_ values. Dickinson and Galazka [53] examined the stability of n-hexadecane in water emulsions stabilized using β-lactoglobulin by measuring d_43_ and d_32_ of the emulsions immediately after preparation and after three weeks of storage. They showed that during storage, the difference between d_43_ and d_32_ values increased significantly due to coalescence, leading to broader distributions.

The difference in droplet size seen in the different samples was attributed to the surfactant located at the O/W interface, which was responsible for emulsion stabilization [51]. As mentioned above, the emulsion gels studied contained four different surface-active molecules that act as emulsifiers in the system: SE, GMS, lecithin, and gelatin. Due to their different molecular size, these surfactants are expected to have different absorption kinetics with respect to the oil/water interface. In general, during homogenization, surfactants with smaller molecular weights will adsorb on the interface faster than larger molecules and will promote the formation of smaller droplets. This stabilization effect is more pronounced in the short term, while in the long term, the larger molecular architectures will usually provide better stabilization [13,54]. In the current research, we focus on the short-term stabilization of small emulsifiers, i.e., SE, lecithin, and GMS, due to the gelation and solidification of the water and oil phases that take place after cooling, which can provide the long-term stabilization effect [55].

In general, all samples consist of similar amounts of lecithin and GMS, whereas the SE type differed. It is assumed that during emulsification, all surfactants present in the formulation are in liquid state and can “compete” for available space at the oil–water interface based on their molecular size and HLB value. The molecular size of SE, which is directly related to its HLB value, is governed by its esterification degree. The SEs used in the current research are commercially available based on the percent of mono-esterified sucrose molecules present in the sample. As specified by the manufacture, SE SP01 is a fully esterified sample, meaning that all eight positions on the sucrose are esterified with palmitic or stearic acids, SE SP02 contains 10% mono-esterified and 90% fully esterified sucrose molecules, while SP30 has 30% mono-esterified and 70% fully esterified sucrose molecules. This product specification leads to various interfacial coverage abilities, whereby during emulsification, mono-esterified sucrose molecules are able to migrate to the interface faster than fully esterified sucrose. Thus, we expect that the SEs will compete for interface positions according to the following order: SP30>SP02>SP01. Generally, SE molecular weight will vary between ≈580 gmol^−1^ for mono-esterified sucrose and ≈2250 gmol^−1^ for fully esterified sucrose (based on palmitic acid). On the other hand, the major constituents of soybean lecithin are phosphatidylcholine, phosphatidyl-ethanolamine, and lysophosphatidylcholine, whose molecular weights range between 800 and 900 gmol^−1^, depending on the fatty acid bound [56]. Therefore, it is assumed that in formulation H1, which contains SP01, the lecithin will reach the oil–water interface faster than the SE, due to its smaller size, and so in this sample, less SE will be present on the oil–water interface, and more SE will be found in the oil phase. The opposite is expected when using SP30, which has a higher content of mono-esterified sucrose that can potentially reach the interface fast and compete with the lecithin for available sites on the interface. As a result, the H6 sample is expected to contain more SE and less lecithin at the oil–water interface compared with H1 and H2 samples. Thus, due to the higher HLB value of lecithin compared with the different SEs, and its higher content in the H6 sample, H6 is expected to stabilize smaller droplets in the O/W bigels, as observed in the micrographs and presented in Table 3. Moreover, the use of SP01 SE by itself, without lecithin, results in unstable emulsions that separate immediately, suggesting that the fully esterified sucrose molecules in SP01 are too big and not amphiphilic enough to stabilize this kind of emulsion gels. It is suspected that due to the high esterification degree of SP01, the steric interference between the molecules is too high, and so, the molecules cannot arrange properly on the interface, leading to de-stabilization. It seems that the SP02 sucrose sample, which contains only 10% mono-esterified sucrose, behaves similar to the fully esterified sample, SP01. Moreover, due to the lower presence of SE in the interface in H1 and H2 bigel systems, more SE is expected to be found in the oil phase compared with the case of H6, thus strengthening the oleogel network within the oil phase. This phenomenon will be further discussed in Section 3.5, Mechanical Properties.

### 3.6. Mechanical Properties

The distribution of the oil droplets in the continuous water phase during the hot emulsification preparation procedure is expected to affect the final mechanical properties of the gel due to various effects such as droplet size, distribution, oleogel hardness, and water/oil interactions. The effect of surfactant type on the final mechanical properties of the bigel was investigated using texture profile analysis (TPA) and spreadability tests.

Table 4 presents the textural attributes calculated from the TPA of the different bigels at room temperature. The results demonstrate hardness values in the order of H1 > H2 > H6, suggesting that surfactant type affects the final mechanical properties of the bigel. A similar trend was observed in the storage modulus values presented in Figure 4 and Table 2. The studied bigels can be referred to as emulsion-filled gels, consisting of structured oil droplets emulsified in a gelatin gel matrix. The mechanical properties of such systems depend on the physiochemical properties of the continuous gel matrix, i.e., gelatin hydrogel, the emulsified structured oil droplets hardness, volume fraction, droplet size and distribution, i.e., oleogel, and the interactions between the two phases [57,58]. According to the bigel preparation procedure, the volume of the oil fraction is constant for all of the samples but according to the microscopy analysis, droplet size, distribution, and content varied among the different samples. Droplet size analysis suggests a negative relationship between droplet size and bigel hardness according to which hardness increases with the decrease in droplet size. Kim et al. [59] studied agar-based emulsion gels with different oil droplet size and found that smaller droplets resulted in harder gels. McClements et al. [60] showed the same trend in their examination of corn oil droplets dispersed in whey protein hydrogel.

Different theories have been developed to describe the effect of particle/droplet type and interfacial relations with continuous gel phase on the final gel properties. Theories introduced by Van der Poel and Kerner suggested that the hardness of the filler, i.e., droplet or particle, affects the hardness of the final gel [61]. Oliver et el. [58] examined different oil phase sources, with different saturated fat contents, in the hydrogel phase, in order to study the effect of oil droplet hardness on the textural characteristics of the emulsion. They found that harder droplets resulted in higher emulsion fracture stress, which implies a harder emulsion gel. To examine this theory, the hardness of an oil phase containing 25 wt % GMS and 2 wt % SEs with different HLB values was measured (Table 4). According to the results, oleogels prepared with SP30 produced harder gels compared with oleogels prepared with SP01. Assuming that oleogels behave as active fillers due to the use of surfactants and, due to the fact that at the studied concentration (1 wt %), gelatin hydrogels produce significantly softer gels compared with the oleogels [19], filler particles deform less than the continuous hydrogel matrix. Thus, the oleogel droplets are expected to have a stronger impact on bigel properties [61]. Our results show the opposite trend, with higher hardness values for bigel systems prepared with the softer oleogel formulation (Table 4). This behavior suggests a stronger effect of droplet size on the bigel’s mechanical properties compared with filler hardness. Another explanation of these results refers to the differences in emulsifier composition at the oil–water interface, resulting in different contents of structuring agent in the oil phase. As discussed above in Section 3.4, we suspect that the water–oil interface in the H6 sample contained higher amounts of SEs compared with the SEs content in the interfaces of the H1 and H2 samples due to the high content of mono-esterified SEs. Therefore, it is assumed that the oil phase in samples H1 and H2 will contain more SEs compared with sample H6. As described previously, SEs can act as oil-structuring agents and lead to oil solidification and higher hardness [34]. Thus, higher SE content in the oil phase of samples H1 and H2 will lead to higher hardness values for the oil phase, resulting in higher bigels hardness.

Cohesiveness values, which describe the internal bonds within the sample or substance that resist mastication before breaks [62], show no significant difference between the formulations. Previous studies done on emulsions prepared using various surfactants with various HLB values concluded that only surfactant concentration, and not the surfactant’s HLB value, has any significant effect on the cohesiveness of the emulsion [63].

The spreadability test results are in line with the TPA with respect to firmness and spreadability (Table 4). Spreadability analysis is performed using a 90-degree cone that penetrates into an equivalent cup filled with the sample. The test provides an indication of the sample’s spreadability in compression mode. Firmness, defined as the maximum force during the penetration, exhibits a trend similar to that determined by the TPA for hardness. The spreadability parameter, defined as the area under the curve of the penetration process, is indicative of how easy it will be to spread the sample: the lower the calculated value, the easier it will be to spread [64]. The results show a positive relationship between sample hardness and spreadability, whereby the hardest formulation, H1, demonstrated the highest spreadability value and the softest formulation, H6, exhibited the lowest spreadability value. The harder bigel exhibited higher loads throughout the entire test, resulting in higher work during cone penetration.

### 3.7. Flow Behavior

**Figure 6:**
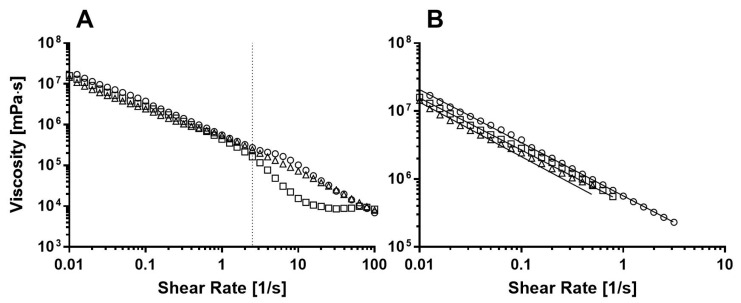
Typical flow obtained for (**A**) different bigel formulations; H1 (о), H2 (∆), and H6 (□), and the (**B**) linear regression of the log-log plot at intermediate shear rates.

Emulsion viscosity is an important characteristic that can influence the production process and properties of the final product (such as the ability to hold air and stabilize foams, and the tendency to separate) [10]. Figure 6 presents the viscosity vs. shear rate curves obtained for the emulsions. The mass fraction of the oil phase is 0.5 (Table 1), and so the volume fraction is higher than 0.5 (due to the higher density of the water phase compared with the oil phase). Such a high-volume fraction results in a flocculated system, in which droplets are clustered and interact with neighboring droplets, as seen in Figure 5. Berli, Quemada, and Parker [65] described such a system as a glassy system, in which droplets are trapped in transient cages formed by their nearest neighbors. Such a system can exhibit both solid-like behavior at low shear rates and liquid-like behavior at high shear rates.

The decrease in viscosity with the increase in shear rate, as observed in Figure 6, defines a pseudoplastic behavior. The most popular model to fit a wide range of shear rates of pseudoplastic systems is the Carreau model [66]

(6) $$\frac{\eta -\eta_{0}}{\eta_{0}-\eta_{\infty }}\;=\;\frac{1}{{\left[1+{\left(\lambda \;\dot{\gamma }\right)}^{2}\right]}^{\left(1-n\right)/2}}$$

where η is the measured viscosity, $\eta_{0}$ is the viscosity at low shear rates, $\eta_{\infty }$ is the viscosity at high shear rates, $\;\dot{\gamma }$ is the shear rate, λ is the relaxation time, and n is the power index. This model describes three main flow regimes according to which a pseudoplastic system will flow. In the initial regime, at very low shear rates, viscosity is constant and equal to $\eta_{0}$. The lower regime shear rate used in the current study, 0.01 s^−1^, was not low enough to detect this regime. In the second regime, at intermediate shear rates, 0.01 to 1 s^−1^, the critical yield stress is exceeded and particles can move past one another, leading to a decrease in the observed viscosity. This region can be seen in Figure 6B and was fitted to a power-law, which can be derived from the Carreau model [67]

(7) $$\eta \;=\;k\times {\;\dot{\gamma }}^{n-1}$$

where k is referred to as consistency index and is the viscosity at zero shear rates [2]. Table 5 presents the fitted parameters. The slope, defined as (n−1), provides n values of 0.20 ± 0.03, 0.19 ± 0.05, and 0.22 ± 0.03 for H1, H2, and H6, respectively. These values support our assumption claiming pseudoplastic behavior, which is characterized by n < 1. Moran-Valero et al. [11] examined the effect of different ratios of lecithin and GMS on the viscosity of O/W emulsions and showed that when lecithin dominates the flow, emulsions exhibit highly pseudoplastic behavior with n = 0.29 [11,67]. On the other hand, when GMS is present in the oil–water interface, Newtonian behavior (n = 1) is observed [68]. They also showed that when the concentration of lecithin is lower than that of GMS, such as in the current studied systems, the rheological behavior is mostly affected by GMS, making the emulsion less pseudoplastic, thus increasing the value of n. The calculated n values in the current research (Table 5) suggest emulsion gel systems in which lecithin dominates the flow; thus, most of the GMS remains in the oil droplets and not in the oil–water interface, as previously suggested by the microscopy analysis (Section 3.4). It is important to note that even though the n value of H6 was found to be statistically different, it is very close to the n values for H1 and H2, and so it can be assumed that the degree of SE esterification did not have a large effect on the pseudoplastic behavior.

Interestingly, a change in the plot slope was seen around shear rate 2.51 s^−1^ (marked by a dashed horizontal line), and this change was more significant for the H6 samples than for the H1 and H2 samples. Such a change in the viscosity slope can imply changes in the flow behavior of the oleogel droplets. More specifically, the H6 curve exhibited a significant decrease in viscosity, which led to an increase in the slope. This sharp decline can be the result of droplet deformation caused by shear forces. In the intermediate shear rates, the velocity gradient is high enough to separate and disrupt the flocs but not high enough as to cause deformation of the droplets. At higher shear rates, the velocity gradient caused by the high shear rate is high enough to deform the droplets; thus, the droplets no longer remain spherical and viscosity decreases more sharply. Otsubo and Prud’homme [69] studied the effects of droplet deformation on viscosity. They claimed that there is a critical droplet deformation index above which the droplets will be broken by the shear forces, and as a result, the coalescence and breakup of droplets will be induced. This breakup and coalescence of droplets results in an increase in droplet size, in turn resulting in a change in the slope of the viscosity curve and in a sharper decrease in viscosity.

According to the third regime, at high shear rates, viscosity reaches a second plateau that is characterized by a constant viscosity and is defined as $\eta_{\infty }$. At these high shear rates, the droplets/particle flocs separate, and the droplets deform and move past one another. This regime was only observed for the H6 sample at shear rates higher than 10 s^−1^. The fact that the third region and the significant slope change were observed for the H6 sample suggests that the droplets in H6 are more sensitive to shear forces, as already shown in the frequency sweep measurements (Figure 4 and Table 2), where H6 bigel exhibited a more frequency-dependent behavior. This phenomenon also supports our assumption that when HLB6 SEs are used, the oil–water interface will contain more SEs compared with the bulk oil, as opposed to bigels with HLB1 and HLB2 SEs, in which SEs are located mainly in the bulk oil, thus strengthening the oleogel network within the oil phase to a lesser degree, resulting in higher sensitivity of the oil droplets to shear forces.

## 4. Conclusions

Bigels formulated using a hot emulsification procedure to mix an oil phase consisting of GMS, various SEs, and lecithin and a water phase containing gelatin and glycerol were investigated. The structure and physiochemical properties of such systems depend on the physiochemical properties of the gelatin gel continuous matrix, the hardness of the emulsified structured GMS/oil droplet, volume fraction, droplet size and distribution, and interface properties. The interface properties are directly related to the interface content of different surfactant molecules. The current research explored the effect on bigel structure and properties of different SEs with different esterification degrees that lead to different HLB values. Rheological and thermal analyses revealed similar gelation-melting transitions for all bigel samples governed by GMS crystallization (at ≈55 °C). The bigel matrix demonstrated physical gel rheological characteristics with higher elastic and solid-like behavior in the H1 and H2 samples, with HLB of 1 and 2, respectively, compared with the H6 sample with HLB 6. A similar trend was also observed in the mechanical analysis with respect to hardness, firmness, and spreadability values in the order of H1 > H2 > H6. This behavior was attributed to the droplet size observed in the microscopy analysis, revealing significantly smaller droplets in the H1 and H2 samples compared with the H6 sample. The difference in droplet size of the different bigels prepared with different SEs was attributed to the diffusion kinetics of the low molecular weight surfactants. More specifically, the ability of mono-esterified SE to diffuse faster than fully esterified SE due to lower molar mass led to a higher content in the O/W interface and lower content in the bulk oil phase. The effect of SEs on the interface was also demonstrated in the flow behavior analysis, where a higher esterification degree leads to higher pseudoplastic behavior. The results demonstrate the importance of the interface content in O/W bigel systems, providing an effective way to alter and control the bigel’s bulk properties.

## Figures and Tables

**Table 1:** Formulation composition of the different bigel samples.

Role	Component	Content (wt %)
Solvent	Water	47
	Oil	35.7
Structuring agents	Gelatin	0.5
	GMS	12.5
Surfactants	Lecithin	0.8
	Sucrose ester *	1
Co-surfactant	Glycerol	2.5
Antifoam agent	Silicon oil	10 ppm

* Sucrose ester SP01, SP10, and SP30 were used to formulate samples H1, H2, and H6, respectively.

**Table 2:** Fit parameters obtained by applying Equations (4) and (5) on the curves presented in Figure 4. Parameters were fitted using three replicates.

Formulation	n′	$G_{0}^{\prime }$	n″	$G_{0}^{\prime \prime }$
H1	0.103 ± 0.005 ^a^	60,950 ± 90 ^a^	0.169 ± 0.008 ^a^	12,300 ± 30 ^a^
H2	0.12 ± 0.02 ^b^	33,900 ± 300 ^b^	0.23 ± 0.03 ^b^	6920 ± 90 ^b^
H6	0.15 ± 0.02 ^c^	23,400 ± 200 ^c^	0.26 ± 0.02 ^c^	5750 ± 50 ^c^

Values in the same column with different letters are significantly different (*p* < 0.05).

**Table 3:** Droplet size analysis performed on the confocal images of the different emulsions (Figure 5) using ImageJ software.

Formulation	d_43_ (µm)	d_32_ (µm)	C_2_
H1	7.84	5.52	0.65
H2	9.77	6.39	0.73
H6	59.42	28.68	1.04

**Table 4:** Mechanical analysis results obtained for the different bigel formulations and oleogels consisting only of the oil phase components.

	Formulation	Hardness bite 1 (N)	Hardness bite 2 (N)	Cohesiveness	Firmness (N)	Spreadability (N mm)
Bigel	H1	11 ± 2 ^a^	6.9 ± 0.7 ^a^	5.7 ± 0.9 ^a^	32 ± 3 ^a^	170 ± 20 ^a^
	H2	11 ± 2 ^a^	6.7 ± 0.6 ^a^	7 ± 2 ^a^	27 ± 2 ^b^	150 ± 10 ^b^
	H6	8 ± 1 ^b^	4.9 ± 0.6 ^b^	6 ± 2 ^a^	21 ± 2 ^c^	130 ± 10 ^c^
Oleogel	SP01	24 ± 1 ^a^	12 ± 1 ^a^	29 ± 1 ^a^		
	SP02	30.9 ± 0.7 ^b^	15.6 ± 0.8 ^a^	30 ± 3 ^a^		
	SP30	47 ± 4 ^c^	30 ± 5 ^b^	60 ± 10 ^b^		

Values in the same column and material type, i.e., bigel and oleogel, with different letters are significantly different (*p* < 0.05). Oleogels prepared using 25 wt %, GMS, 2 wt % SEs (different types), and canola oil.

**Table 5:** Power-law fit parameters obtained from the linear regression of the log-log plot at low shear rate for the different bigel samples. Parameters were fitted using three replicates.

Formulation	K (mPa·s^n^)	n
H1	(6.35 ± 0.05) × 10^5 a^	0.20 ± 0.03 ^a^
H2	(4.22 ± 0.07) × 10^5 b^	0.19 ± 0.05 ^a^
H6	(4.27 ± 0.04) × 10^5 b^	0.22 ± 0.03 ^b^

Values in the same column with different letters are significantly different (*p* < 0.05).

## References

[1] Yukuyama M.N., Ghisleni D.D.M., Pinto T.J.A., Bou-Chacra N.A. (2016). Nanoemulsion: Process selection and application in cosmetics—A review. Int. J. Cosmet. Sci..

[2] McClements D.J. (2016). Food Emulsions: Principles, Practices, and Techniques.

[3] Patel A.R., Dewettinck K. (2016). Edible oil structuring: An overview and recent updates. Food Funct..

[4] Rehman K., Mohd Amin M.C.I., Zulfakar M.H. (2014). Development and Physical Characterization of Polymer-Fish Oil Bigel (Hydrogel/Oleogel) System as a Transdermal Drug Delivery Vehicle. J. Oleo Sci..

[5] Ibrahim M.M., Hafez S.A., Mahdy M.M. (2013). Organogels, hydrogels and bigels as transdermal delivery systems for diltiazem hydrochloride. Asian J. Pharm. Sci..

[6] Lupi F.R., Shakeel A., Greco V., Oliviero Rossi C., Baldino N., Gabriele D. (2016). A rheological and microstructural characterisation of bigels for cosmetic and pharmaceutical uses. Mater. Sci. Eng. C.

[7] Gonzalez-Gutierrez J., Scanlon M.G., Marangoni A. (2018). Chapter 5—Rheology and Mechanical Properties of Fats. Structure-Function Analysis of Edible Fats.

[8] Wakhet S., Singh V.K., Sahoo S., Sagiri S.S., Kulanthaivel S., Bhattacharya M.K., Kumar N., Banerjee I., Pal K. (2015). Characterization of gelatin–agar based phase separated hydrogel, emulgel and bigel: A comparative study. J. Mater. Sci. Mater. Med..

[9] Singh V.K., Banerjee I., Agarwal T., Pramanik K., Bhattacharya M.K., Pal K. (2014). Guar gum and sesame oil based novel bigels for controlled drug delivery. Colloids Surfaces B Biointerfaces.

[10] Hasenhuettl G.L., Hartel R.H. (2011). Food Emulsifiers and Their Applications.

[11] Moran-Valero M.I., Ruiz-Henestrosa V.M.P., Pilosof A.M.R. (2017). Synergistic performance of lecithin and glycerol monostearate in oil/water emulsions. Colloids Surfaces B Biointerfaces.

[12] Dickinson E. (2009). Hydrocolloids as emulsifiers and emulsion stabilizers. Food Hydrocoll..

[13] McClements D.J., Jafari S.M. (2018). Improving emulsion formation, stability and performance using mixed emulsifiers: A review. Adv. Colloid Interface Sci..

[14] ICI Americas, Inc. (1984). The HLB System A Time-Saving Guide to Emulsifier Selection.

[15] Ojeda-Serna I.E., Rocha-Guzmán N.E., Gallegos-Infante J.A., Cháirez-Ramírez M.H., Rosas-Flores W., Pérez-Martínez J.D., Moreno-Jiménez M.R., González-Laredo R.F. (2019). Water-in-oil organogel based emulsions as a tool for increasing bioaccessibility and cell permeability of poorly water-soluble nutraceuticals. Food Res. Int..

[16] Pichot R., Spyropoulos F., Norton I.T. (2010). O/W emulsions stabilised by both low molecular weight surfactants and colloidal particles: The effect of surfactant type and concentration. J. Colloid Interface Sci..

[17] Samui T., Goldenisky D., Rosen-Kligvasser J., Davidovich-Pinhas M. (2020). The development and characterization of novel in-situ bigel formulation. Food Hydrocoll..

[18] Karim A.A., Bhat R. (2009). Fish gelatin: Properties, challenges, and prospects as an alternative to mammalian gelatins. Food Hydrocoll..

[19] Ross-Murphy S.B. (1992). Structure and rheology of gelatin gels: Recent progress. Polymer.

[20] Ojijo N.K.O., Neeman I., Eger S., Shimoni E. (2004). Effects of monoglyceride content, cooling rate and shear on the rheological properties of olive oil/monoglyceride gel networks. J. Sci. Food Agric..

[21] Ferro A.C., Okuro P.K., Badan A.P., Cunha R.L. (2019). Role of the oil on glyceryl monostearate based oleogels. Food Res. Int..

[22] Van Nieuwenhuyzen W., Tomás M.C. (2008). Update on vegetable lecithin and phospholipid technologies. Eur. J. Lipid Sci. Technol..

[23] Klang V., Valenta C. (2011). Lecithin-based nanoemulsions. J. Drug Deliv. Sci. Technol..

[24] Szűts A., Szabó-Révész P. (2012). Sucrose esters as natural surfactants in drug delivery systems—A mini-review. Int. J. Pharm..

[25] Zou Y., Pan R., Wan Z., Guo J., Wang J., Yang X. (2017). Gel-like emulsions prepared with zein nanoparticles produced through phase separation from acetic acid solutions. Int. J. Food Sci. Technol..

[26] Alam M.M., Aramaki K. (2009). Glycerol effects on the formation and rheology of hexagonal phase and related gel emulsion. J. Colloid Interface Sci..

[27] Kulkarni R.D., Goddard E.D., Kanner B. (1977). Mechanism of Antifoaming: Role of Filler Particle. Ind. Eng. Chem. Fundam..

[28] Zhang D., Mu T., Sun H. (2017). Calorimetric, rheological, and structural properties of potato protein and potato starch composites and gels. Starch/Staerke.

[29] Ravanagh G.M., Ross-Murphy S.B., Kavanagh G.M., Ross-Murphy S.B. (1998). Rheological characterization of polymer gels. Prog. Polym. Sci..

[30] Sun X.D., Arntfield S.D. (2011). Gelation properties of salt-extracted pea protein isolate induced by heat treatment: Effect of heating and cooling rate. Food Chem..

[31] Chen C.C.-H., Terentjev E.M., Marangoni A.G., Garti N. (2018). Monoglycerides in oils. Edible Oleogels: Structure and Health Implications.

[32] Tosh S.M., Marangoni A.G. (2004). Determination of the maximum gelation temperature in gelatin gels. Appl. Phys. Lett..

[33] López-Martínez A., Morales-Rueda J.A., Dibildox-Alvarado E., Charó-Alonso M.A., Marangoni A.G., Toro-Vazquez J.F. (2014). Comparing the crystallization and rheological behavior of organogels developed by pure and commercial monoglycerides in vegetable oil. Food Res. Int..

[34] Bin Sintang M.D., Danthine S., Patel A.R., Rimaux T., Van De Walle D., Dewettinck K. (2017). Mixed surfactant systems of sucrose esters and lecithin as a synergistic approach for oil structuring. J. Colloid Interface Sci..

[35] Ghan S.Y., Siow L.F., Tan C.P., Cheong K.W., Thoo Y.Y. (2020). Influence of Soya Lecithin, Sorbitan and Glyceryl Monostearate on Physicochemical Properties of Organogels. Food Biophys..

[36] Wolfson A., Dlugy C., Shotland Y. (2007). Glycerol as a green solvent for high product yields and selectivities. Environ. Chem. Lett..

[37] Vaclavik V.A., Christian E.W., Vaclavik V.A., Christian E.W. (2008). Water. Essentials of Food Science.

[38] Bellich B., Borgogna M., Cok M., Cesàro A. (2011). Water evaporation from gel beads: A calorimetric approach to hydrogel matrix release properties. J. Therm. Anal. Calorim..

[39] Wang R., Wang Q., Li L. (2003). Evaporation behaviour of water and its plasticizing effect in modified poly(vinyl alcohol) systems. Polym. Int..

[40] Goodrum J.W. (2002). Volatility and boiling points of biodiesel fromvegetable oils and tallow. Biomass Bioenergy.

[41] Hernandez-Izquierdo V.M., Krochta J.M. (2008). Thermoplastic Processing of Proteins for Film Formation—A Review. J. Food Sci..

[42] Shankar S., Teng X., Li G., Rhim J.-W. (2015). Preparation, characterization, and antimicrobial activity of gelatin/ZnO nanocomposite films. Food Hydrocoll..

[43] Szuts A., Budai-Szucs M., Eros I., Otomo N., Szabó-Révész P. (2010). Study of gel-forming properties of sucrose esters for thermosensitive drug delivery systems. Int. J. Pharm..

[44] Moreno H.M., Domínguez-Timón F., Díaz M.T., Pedrosa M.M., Borderías A.J., Tovar C.A. (2020). Evaluation of gels made with different commercial pea protein isolate: Rheological, structural and functional properties. Food Hydrocoll..

[45] Ross-Murphy S.B. (1995). Rheological characterization of physical gels 1. J. Texture Stud..

[46] Rosalina I., Bhattacharya M. (2002). Dynamic rheological measurements and analysis of starch gels. Carbohydr. Polym..

[47] Campo L., Tovar C. (2008). Influence of the starch content in the viscoelastic properties of surimi gels. J. Food Eng..

[48] Sadecka E., Szela̧g H. (2013). One-step synthesis of W/O and O/W emulsifiers in the presence of surface active agents. J. Surfactants Deterg..

[49] Stauffer C.E. (1999). Emulsifiers.

[50] Franco J.M., Guerrero A., Gallegos C. (1995). Rheology and processing of salad dressing emulsions. Rheol. Acta.

[51] Knoth A., Scherze I., Muschiolik G. (2005). Stability of water-in-oil-emulsions containing phosphatidylcholine-depleted lecithin. Food Hydrocoll..

[52] Surh J., Decker E.A., McClements D.J. (2006). Properties and stability of oil-in-water emulsions stabilized by fish gelatin. Food Hydrocoll..

[53] Dickinson E., Galazka V.B. (1991). Emulsion stabilization by ionic and covalent complexes of β-lactoglobulin with polysaccharides. Top. Catal..

[54] Yang Y., Leser M.E., Sher A.A., McClements D.J. (2013). Formation and stability of emulsions using a natural small molecule surfactant: Quillaja saponin (Q-Naturale^®^). Food Hydrocoll..

[55] Surh J., Vladisavljević G.T., Mun S., McClements D.J. (2007). Preparation and characterization of water/oil and water/oil/water emulsions containing biopolymer-gelled water droplets. J. Agric. Food Chem..

[56] Dickinson E. (1993). Towards more natural emulsifiers. Trends Food Sci. Technol..

[57] Vliet T. (1988). Rheological properties of filled gels. Influence of filler matrix interaction. Colloid Polym. Sci..

[58] Oliver L., Scholten E., van Aken G.A. (2015). Effect of fat hardness on large deformation rheology of emulsion-filled gels. Food Hydrocoll..

[59] Kim K.-H., Gohtani S., Yamano Y. (1996). Effects of Oil Droplets on Physical and Sensory Properties of O/W Emulsion Agar Gel. J. Texture Stud..

[60] Mcclements D.J., Monahan F.J., Kinsella J.E. (1993). Effect of Emulsion Droplets on the Rheology of Whey Protein Isolate Gels. J. Texture Stud..

[61] Sala G., Van Aken G.A., Stuart M.A.C., Van De Velde F. (2007). Effect of droplet-matrix interactions on large deformation properties of emulsion-filled gels. J. Texture Stud..

[62] Alina Surmacka S. (2002). Texture is a sensory property. Food Qual. Prefer..

[63] Lemaitre-Aghazarian V., Piccerelle P., Reynier J.P., Joachim J., Phan-Tan-Luu R., Sergent M. (2004). Texture optimization of water-in-oil emulsions. Pharm. Dev. Technol..

[64] Rohm H., Strobl M., Jaros D. (1997). Butter colour affects sensory perception of spreadability. Eur. Food Res. Technol..

[65] Berli C.L.A., Quemada D., Parker A. (2003). Gel transition of depletion flocculated emulsions. Colloids Surfaces A Physicochem. Eng. Asp..

[66] Barnes H.A. (1996). Rheology: Principles, Measurements and Applications.

[67] Bhattacharya S., Shylaja M.H., Manjunath M.S., Sankar U. (1998). Rheology of lecithin dispersions. JAOCS J. Am. Oil Chem. Soc..

[68] Mao L., Calligaris S., Barba L., Miao S. (2014). Monoglyceride self-assembled structure in O/W emulsion: Formation, characterization and its effect on emulsion properties. Food Res. Int..

[69] OTSUBO Y., PRUD’HOMME R.K. (1992). Flow Behavior of Oil-in-Water Emulsions. Nihon Reoroji Gakkaishi (J. Soc. Rheol. Jpn.).

